# Coated Blade Spray Ion Mobility Spectrometry

**DOI:** 10.1021/acs.analchem.3c05586

**Published:** 2024-02-13

**Authors:** Christian Thoben, Jannie J. Stadtler, Paul R. Simon, Christian-Robert Raddatz, Merle Sehlmeyer, Stefan Zimmermann

**Affiliations:** Institute of Electrical Engineering and Measurement Technology, Department of Sensors and Measurement Technology, Leibniz University Hannover, Appelstraße 9A, 30167 Hannover, Germany

## Abstract

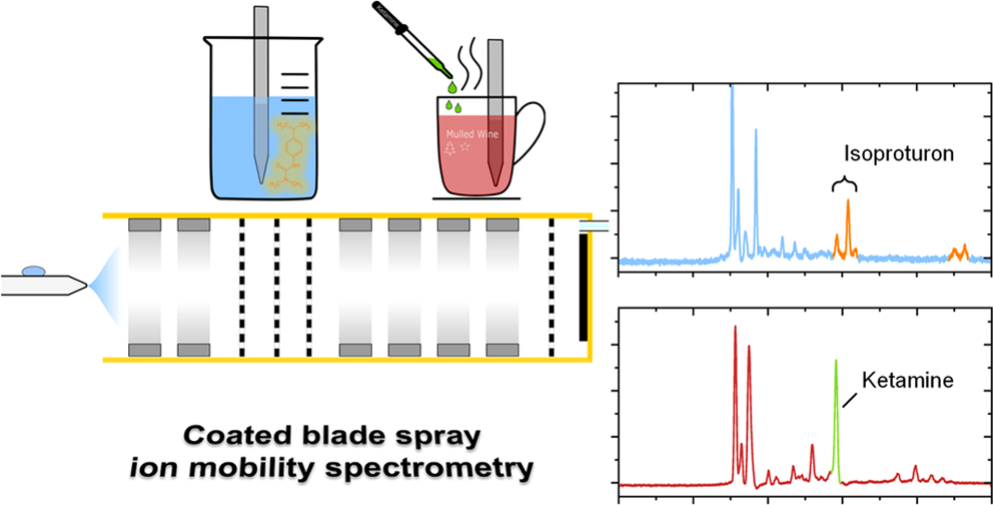

Coated blade spray (CBS) is a microextraction technology with blades that serve as both the extraction device and the electrospray ionization (ESI) emitter. CBS is designed for easy and rapid extraction of analytes in complex matrices as well as ESI directly from the blade. The technology selectively enriches the components of interest on a coated metal blade. The coating consists of a selective polymer. So far, CBS has only been coupled with mass spectrometry but never with ion mobility spectrometry (IMS), where ions are separated and detected based on their ion mobility in a drift gas under the influence of an electric field, while instrumentation is compact and easy to operate so that the advantages of CBS can be particularly well exploited. Therefore, this work focuses on coupling CBS with our previously described ESI-IMS. The ion mobility spectrometer has a drift length of only 75 mm and provides a high resolving power of *R*_P_ = 100. In this work, preliminary measurements of CBS-IMS are presented. In particular, the detection of benzodiazepines and ketamine in drinks and the pesticide isoproturon in water samples is shown to demonstrate the feasibility of CBS-IMS.

## Introduction

Ion mobility spectrometry (IMS) characterizes and separates ions through their specific mobility in a neutral gas under the influence of an electric field.^1^ Currently, IMS is increasingly used in various applications with gaseous and liquid samples. IMS for field applications is usually operated at ambient pressure, so vacuum pumps are not required, and the instrumental complexity is low compared to mass spectrometry (MS). In addition, ion mobility spectrometers can be built with low cost and compactness,^2−4^ including 3D-printed drift tubes,^5,6^ and the operation of IMS is cost-effective and conserves resources.^7^ Furthermore, IMS provides short drift times in the millisecond range and therefore relatively fast analyzing times. IMS can be used for the detection of drugs,^1,8^ explosives,^1,9^ pesticides, or other contaminants.^1,10^

Electrospray ionization is often used for liquid samples, transferring the analytes to the gas phase and ionizing the analytes at the same time. An electrospray is a dispersed nebula of charged droplets generated by a liquid sample emitted from a capillary under the influence of a strong electric field. Eventually, gas-phase ions are generated in a complex process induced by solvent evaporation and droplet jet fissions due to coulombic stress.^11^

However, electrospray ionization also has its drawbacks. For example, in complex samples, signal suppression might be possible due to different surface activities or suppression from salts or surfactants.^12^ Furthermore, water samples without any additional solvents are hard to electrospray due to the high surface tension of water. For this reason, the most commonly used electrospray solvents consist of methanol/water or acetonitrile/water mixtures. This leads to dilution of water samples, but the signal response is also reduced at high water content.^13,14^

Therefore, high-performance liquid chromatography (HPLC) is a suitable technique for the analysis of water samples, whereby the instrumental effort is increased by another technique. Large instruments, pumps, and other accessories are needed. In addition, large amounts of solvents are consumed and the analysis time is considerably longer, unless HPLC is also miniaturized.^7,15^ Furthermore, sample preparation may be necessary with HPLC. Possibilities for analyzing samples without time-consuming sample preparation include solid-phase microextraction (SPME) or the paper spray method. In the latter, the sample is applied to a cut piece of paper, dried, and then mixed with a small amount of solvent and applied to a high voltage similar to electrospray ionization. Used in combination with IMS, detection limits in the mg/L range can be achieved.^16,17^

Currently, paper spray is being further developed in research to ensure selective extraction of analytes. One example is the immobilization of aptamers^18,19^ or the use of a molecularly imprinted polymer.^20^ However, due to the nonuniform fibers at the tip, paper spray can form multiple spray jets, which is disadvantageous in terms of reproducibility.^21^

## Coated Blade Spray

Another approach without time-consuming sample preparation is provided by the coated blade spray (CBS) technology introduced by Pawliszyn et al.^22−25^ The CBS technology is based on SPME with the extraction device being the electrospray ionization emitter. In SPME, a small amount of the extracting phase dispersed on a solid support is exposed to the sample for a well-defined period of time. A partitioning equilibrium between the sample matrix and the extraction phase is reached after an equilibrium extraction time. Before this time, the amount of analyte extracted is related to time.^26^ Furthermore, the amount of analyte extracted by the extraction device is proportional to its free concentration in the sample to a first approximation.^27^ The volume of the extraction phase is particularly important. A thin coating with a large surface area, as is the case with CBS, ensures rapid sampling and, more importantly, rapid desorption.^24^

The coated blade acts as an extraction device and electrospray ionization emitter. The overall analytical process includes extraction from a complex untreated matrix, rinsing of the blade, desorption and ionization, and in this case separation and detection via IMS. The extraction method allows the analytes to be enriched directly from various media.^23,24^ In CBS, the components of interest are selectively enriched on a coated metal blade. The coating consists of a selective polymer.^22,25^ The enrichment indeed has a positive effect on the detection limit, which is particularly interesting when IMS is used as a separation method and detector, for example, for point-of-care applications. Furthermore, the rigid and robust blades allow for easy sample collection as well as easy handling and automation of the measurement process. In addition, the blades can also be reused.^23^

The following is a brief description of the analytical process; a detailed report for using CBS can be found elsewhere.^22,25^ First, the blade is preconditioned with a mixture of 50% methanol, 25% acetonitrile, and 25% isopropanol. Then, the blade is held or preferably stirred for a defined extraction time in the extraction matrix to extract and ideally enrich the analytes by the extraction phase coating on the blade. Extraction is followed by a short rinse with water to remove unwanted matrix components from the blade. The prepared dried blade is clamped to an autosampler, a desorption solvent drop is added, and high voltage is applied. This desorbs the analyte molecules from the blade and ionizes the analytes via electrospray. In CBS, ionization runs under ambient conditions. Subsequently, the analyte ions are separated and detected with IMS.

To summarize, CBS is designed for simple and rapid extraction of analytes in complex matrices as well as for electrospray ionization directly from the blade.

So far, CBS has only been reported in combination with mass spectrometry but never with IMS. Therefore, to the best of our knowledge, we present the first coupling of CBS and IMS. In particular, the advantages of CBS can be well exploited in IMS. In this work, we focus on coupling CBS with our previously described ESI-IMS.^28,29^ The ion mobility spectrometer has a drift length of only 75 mm and provides a high resolving power of *R*_P_ = 100. Here, a first characterization of CBS-IMS is presented. In particular, the detection of benzodiazepines and ketamine in drinks and the pesticide isoproturon in water samples is shown in preliminary measurements to demonstrate the feasibility of CBS-IMS.

## Ion Mobility Spectrometry

IMS separates ions through their specific mobility in a neutral gas under the influence of an electric field. The ion mobility *K* depends, among other things, on the charge of the ions, their mass, and the collision cross-section between ions and neutral particles, with the collision cross-section being the most important parameter. In order to correct the influence of temperature and pressure, the reduced ion mobility *K*_0_ is introduced, leading to a certain comparability between different setups.^30^ The reduced ion mobility *K*_0_ can be calculated according to eq 1.^1^
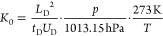
1where *L*_D_ corresponds to the length of the drift region in cm, *t*_D_ corresponds to the drift time of the ion species in s, *U*_D_ corresponds to the applied drift voltage in V, *p* corresponds to the internal pressure of the ion mobility spectrometer in hPa, and *T* corresponds to the temperature in the drift region in Kelvin. The ion mobility spectrum is usually represented either by the drift time of the ions, namely, the time the ions need to pass through the drift region, or by the reduced ion mobility.

One commonly used benchmark for the performance of an ion mobility spectrometer is the resolving power, which is defined according to eq 2.^1,31^

Resolving powers over 80 are considered to be high-resolution ion mobility spectrometers.^32,33^
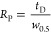
2While resolving power does not explicitly define separation capacity, in linear IMS as discussed here, it is proportional to the metric of resolution or how well two peaks are resolved.^34^ Thus, maximizing the resolving power is a common goal in IMS instrument design.

## Experimental Section

**Figure 1:**
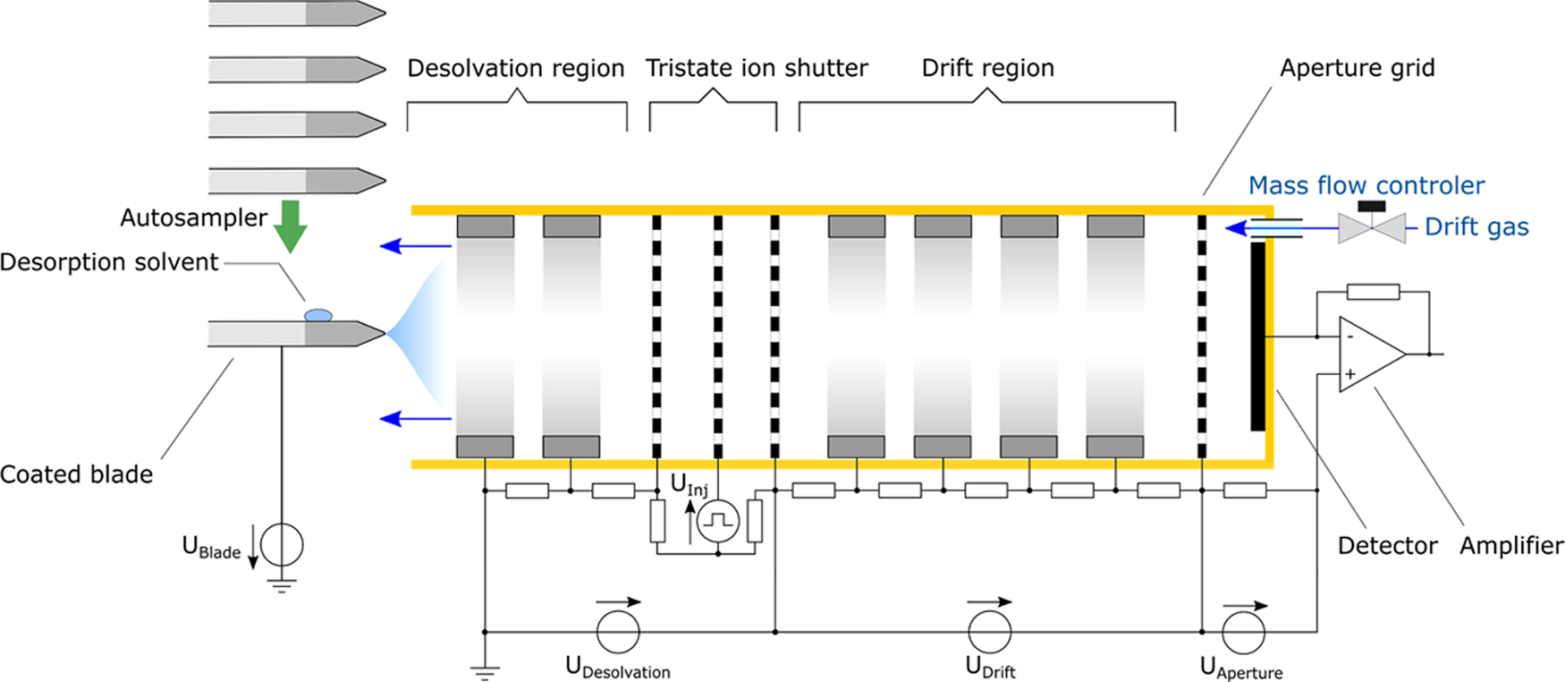
Schematic of the CBS-IMS device with the voltages for the coated blade spray, the desolvation region, the drift region, and the aperture grid as well as the pulsed voltage at the ion gate. The autosampler, which positions the blades, adds a droplet with defined volume of the desorption solvent to the coated area of the blade, and provides the connection to the high voltage, is sketched. The controlled mass flow of the drift gas with the drift gas outlet at the beginning of the desolvation region is also shown.

**Figure 2:**
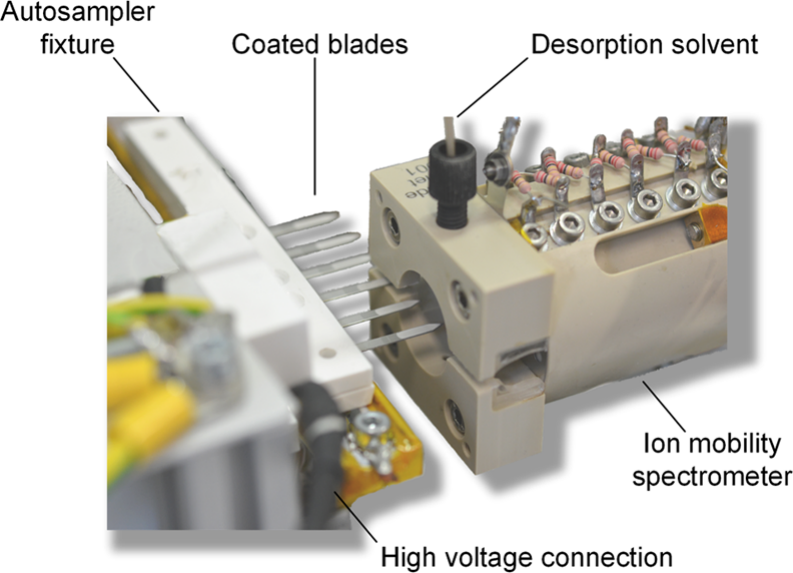
Photo of the coated blades clamped to the autosampler and placed in front of the ion mobility spectrometer. The autosampler also controls the application of one droplet with a defined volume of the desorption solvent to the coated area of the blade.

In this work, we used our ion mobility spectrometer with 75 mm drift tube length. A detailed description can be found elsewhere.^28,29^ Here, the ions are generated by electrospray ionization directly from the coated blades (CB-HLB blades, Restek GmbH, Bad Homburg, Germany) within a desolvation region of 50 mm length. The coated blades are made of stainless steel and are 42 mm long and 10 mm of the tip is coated with a hydrophilic–lipophilic balanced (HLB) sorbent. The samples are extracted from different extraction matrices by the coated blades. The sample solvent is either a mixture of 90% water and 10% methanol or, in the case of application measurements, 80% water and 20% mulled wine, respectively, 100% real water samples. To provide higher throughput, up to 10 blades can be sequentially positioned in front of the ion mobility spectrometer by an autosampler (PAS Technology Deutschland GmbH, Magdala, Germany). The autosampler also adds a defined volume of the desorption solvent to the coated area before turning on the ESI voltage; here, we use 20 μL of a mixture of 20% water, 80% methanol, and an addition of 0.1% formic acid. This desorption solvent is used for every measurement in this work. The ESI voltage of 3 to 4 kV is applied between the coated blades and the grounded first ring of the inlet of the desolvation region. Consequently, the detector and the analog-to-digital converter are at high potential.^35^ The voltages across the desolvation region and the drift region are supplied by a 12.5 kV power supply from FuG Elektronik GmbH (Schechen, Germany). A self-designed and self-constructed amplifier with low noise is used as transimpedance amplifier.^36^Table 1 gives an overview of the relevant operating parameters of the CBS-IMS device and an instrumental diagram is illustrated in Figure 1. A photograph of this arrangement is shown in Figure 2.

**Table 1:** CBS-IMS Operating Parameters

parameter	value	parameter	value
length of drift region	75 mm	drift field strength	60 V/mm
length of desolvation region	50 mm	desolvation field strength	60 V/mm
blade-to-inlet voltage	3–4 kV	desorption solvent	20:80 water/methanol + 0.1% formic acid
drift gas flow rate	250 mL/min	desorption solvent volume	20 μL
drift gas dew point	–85 °C	drift region temperature	22–25 °C
drift gas	nitrogen	desolvation region temperature	22–25 °C
pressure (absolute)	998–1021 hPa		

### Chemicals

LC-MS-grade water, methanol (MeOH), and acetonitrile were purchased from Altmann Analytik GmbH & Co. KG, Germany. LC-MS-grade isopropanol, the herbicide isoproturon (analytical standard), the benzodiazepines diazepam (analytical standard) and midazolam (analytical standard), and the drug ketamine (analytical standard) were purchased from Sigma-Aldrich Chemie GmbH, Germany. The test solutions were prepared at a concentration of 1 μg/L to 8 mg/L. As a sample solvent for the extraction matrix, LC-MS-grade water and MeOH, mulled wine (“Heißer Hirsch”, Acht Grad plus GmbH, Germany), tap water (Hannover, Germany), stream water (Mühlenbach, Neukloster, Germany), spring water (Rosenborn, Harsefeld, Germany), and river water (Leine, Hannover, Germany) are used.

## Results and Discussion

### Extraction Times

**Figure 3:**
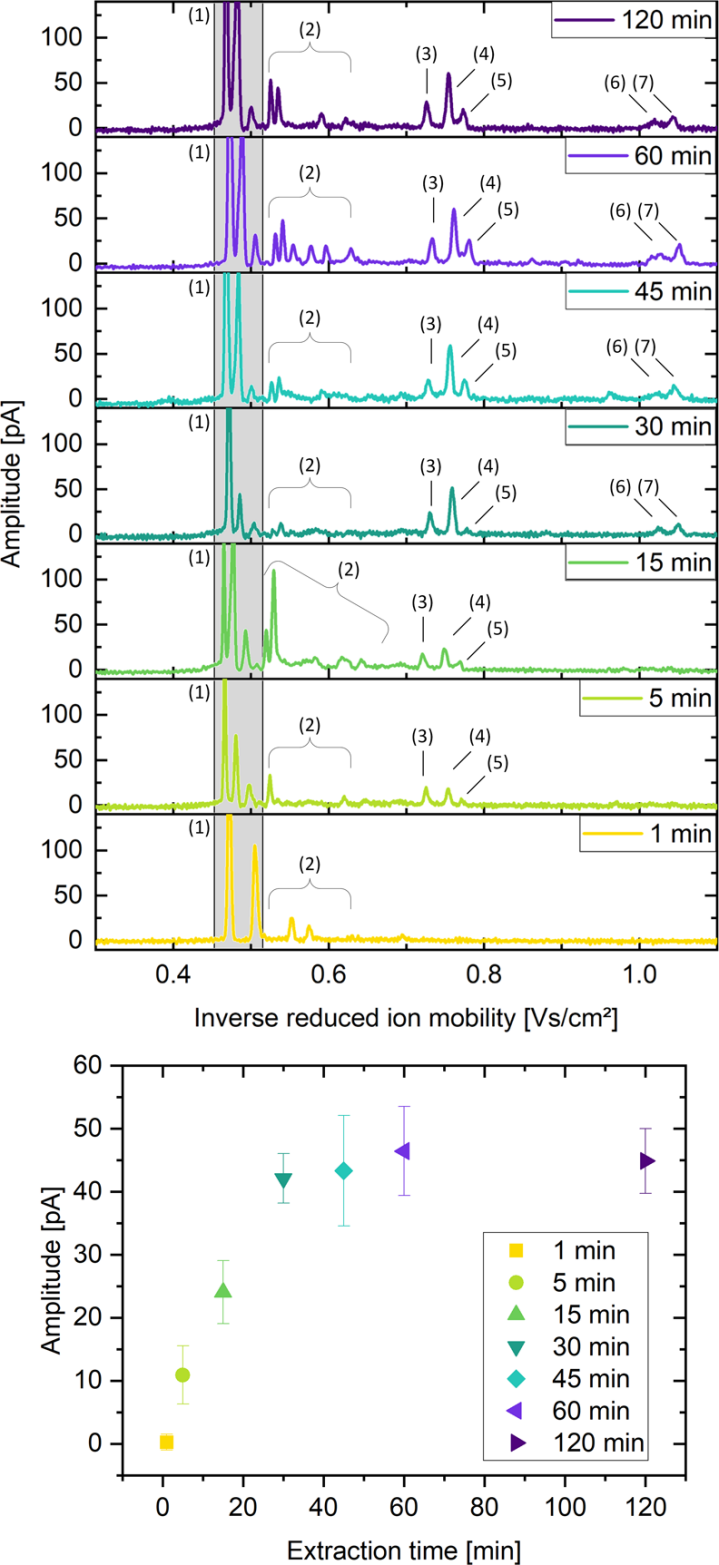
Ion mobility spectra of a sample solution of 25 μg/L isoproturon in 90:10 H_2_O/MeOH for different extraction times extracted via coated blades and detected via CBS-IMS (top) and peak amplitude of isoproturon for different extraction times (bottom). The peaks in the spectrum can be assigned as follows: solvent peaks (1) at *K*_0_ = 2.13, 2.07, and 2.00 cm^2^ V^–1^ s^–1^, background peaks (2) between *K*_0_ = 1.93 and 1.56 cm^2^ V^–1^ s^–1^, protonated isoproturon monomer peak (3) at *K*_0_ = 1.37 cm^2^ V^–1^ s^–1^, sodium-bound isoproturon monomer (4) at *K*_0_ = 1.32 cm^2^ V^–1^ s^–1^, and an unknown isoproturon monomer adduct (5) at *K*_0_ = 1.29 cm^2^ V^–1^ s^–1^ as well as a protonated isoproturon dimer (6) at *K*_0_ = 0.98 cm^2^ V^–1^ s^–1^ and sodium-bound isoproturon dimer (7) at *K*_0_ = 0.96 cm^2^ V^–1^ s^–1^.

In a first step, preliminary measurements were performed, and the influence of different extraction times while stirring the blade in the sample for 1–120 min was investigated. For this purpose, isoproturon with a concentration of 25 μg/L was used. Figure 3 shows the relationship between the extraction time and the amplitude of the recorded signal in the ion mobility spectrum. For an extraction time of 1 min, not enough analyte molecules could be enriched to form a visible analyte peak. As the extraction time increases, the amount of analyte extracted increases, which is observed as an increase in the ion intensity measured by the ion mobility spectrometer, which can be assigned according to our previous work^37^ as protonated isoproturon monomer at a reduced ion mobility of *K*_0_ = 1.37 cm^2^ V^–1^ s^–1^, sodium-bound isoproturon monomer at *K*_0_ = 1.32 cm^2^ V^–1^ s^–1^, and an unknown isoproturon monomer adduct at *K*_0_ = 1.29 cm^2^ V^–1^ s^–1^, which presumably forms from substances present in the blade. For extraction times longer than 30 min, saturation is reached, and also, the protonated isoproturon dimer at *K*_0_ = 0.98 cm^2^ V^–1^ s^–1^ and sodium-bound isoproturon dimer at *K*_0_ = 0.96 cm^2^ V^–1^ s^–1^ start to form. In addition to the solvent peaks at *K*_0_ = 2.13, 2.07, and 2.00 cm^2^ V^–1^ s^–1^, background peaks appear between *K*_0_ = 1.93 and 1.56 cm^2^ V^–1^ s^–1^, which are presumably formed by substances present in the blade.

An extraction time of 5 min seems to be a good compromise between time expenditure and signal intensity, so it was chosen for all following measurements.

### Extraction and Separation Performance

**Figure 4:**
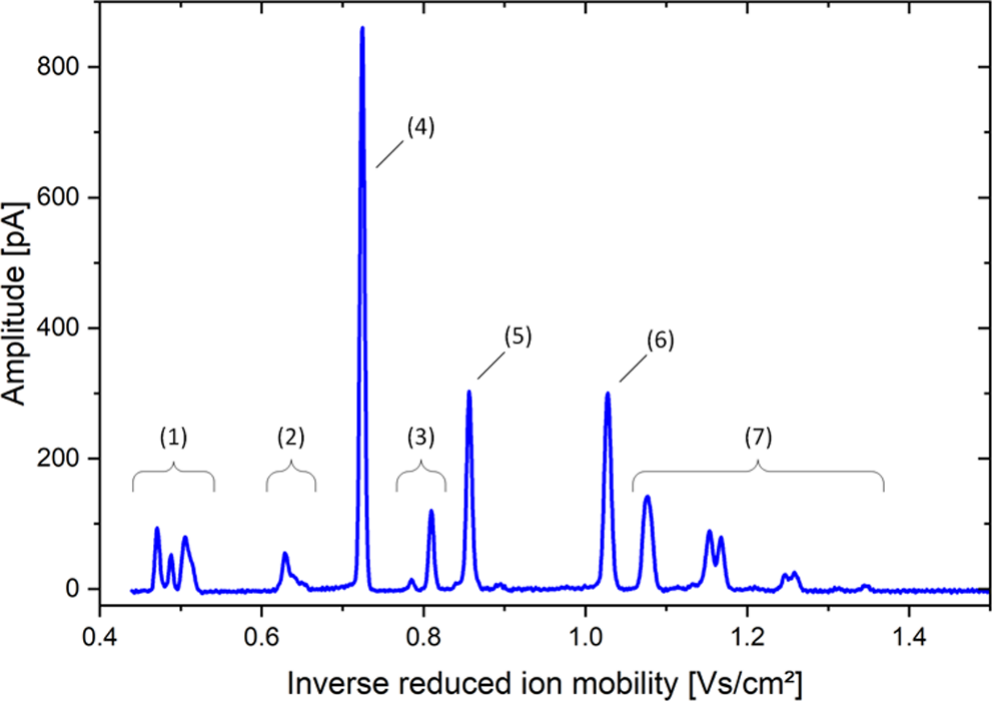
Ion mobility spectrum of 90:10 H_2_O/MeOH spiked with 3 mg/L ketamine, 3 mg/L diazepam, and 3 mg/L midazolam extracted via coated blades and detected via CBS-IMS. The peaks in the spectrum can be assigned as follows: solvent peaks (1) between *K*_0_ = 2.12 and 1.98 cm^2^ V^–1^ s^–1^ and background peaks (2) between *K*_0_ = 1.59 and 1.54 cm^2^ V^–1^ s^–1^ and background peaks (3) at *K*_0_ = 1.27 and 1.24 cm^2^ V^–1^ s^–1^ as well as the ketamine peak (4) at *K*_0_ = 1.38 cm^2^ V^–1^ s^–1^ and a resolving power of *R*_P_ = 110, the diazepam peak (5) at *K*_0_ = 1.17 cm^2^ V^–1^ s^–1^ with *R*_P_ = 113, the midazolam peak (6) at *K*_0_ = 0.97 cm^2^ V^–1^ s^–1^ with *R*_P_ = 105, and the dimer peaks between *K*_0_ = 0.93 and *K*_0_ = 0.74 cm^2^ V^–1^ s^–1^.

After choosing the extraction time of 5 min, the extraction performance of the coated blades and the separation performance of the ion mobility spectrometer were evaluated with a mixture of two benzodiazepines and the drug ketamine. Figure 4 shows the ion mobility spectrum with the calculated reduced ion mobility according to eq 1 of 90:10 H_2_O/MeOH spiked with 3 mg/L ketamine, 3 mg/L diazepam, and 3 mg/L midazolam. The peaks of ketamine (*K*_0_ = 1.38 cm^2^ V^–1^ s^–1^, *R*_P_ = 110), diazepam (*K*_0_ = 1.17 cm^2^ V^–1^ s^–1^, *R*_P_ = 113), and midazolam (*K*_0_ = 0.97 cm^2^ V^–1^ s^–1^, *R*_P_ = 105) are well separated. Thus, the blades extract all three compounds, and our coated blade spray emitter efficiently ionizes the compounds. Furthermore, a good sensitivity and resolving power of the CBS-IMS device can be expected by looking at this spectrum.

### Limits of Detection

**Figure 5:**
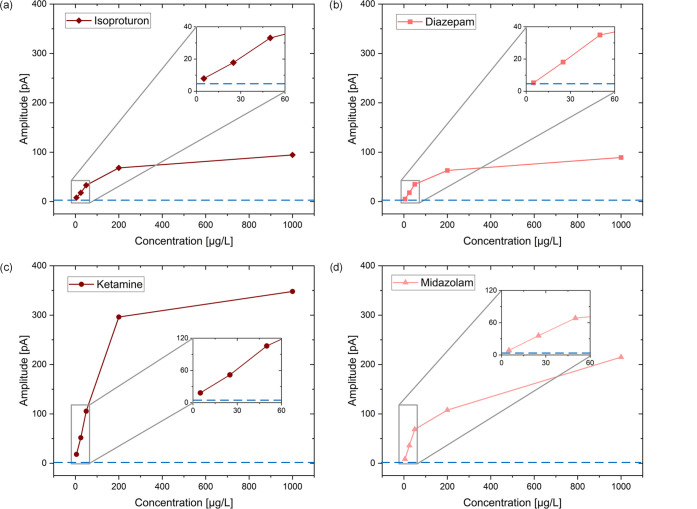
Calibration curves of the herbicide isoproturon (a), diamonds, the drug ketamine (c), circles, and the benzodiazepines diazepam (b), squares, and midazolam (d), triangles, each dissolved in 90:10 H_2_O/MeOH, with an extraction time of only 5 min, extracted via coated blades and detected via CBS-IMS.

In Figure 5, the detection limits for the used analytes were roughly estimated for the chosen extraction time of 5 min. Therefore, the first three data points of the calibration curves were used for a linear regression, giving the sensitivity in pA/μg L^–1^. The intersection of this linear regression with 3 times the standard deviation of the blank noise in pA gives the detection limits in μg/L. The setup provides good detection limits with 5.3 μg/L for isoproturon, 5 μg/L for diazepam, 2.6 μg/L for midazolam, and 1.7 μg/L for ketamine. At higher concentrations, all signals saturate. However, as shown above, longer extraction times could further improve the detection limits, since the calibration curves would show better sensitivity, especially in the low concentration range.

### Application Measurements of Complex Matrices

**Figure 6:**
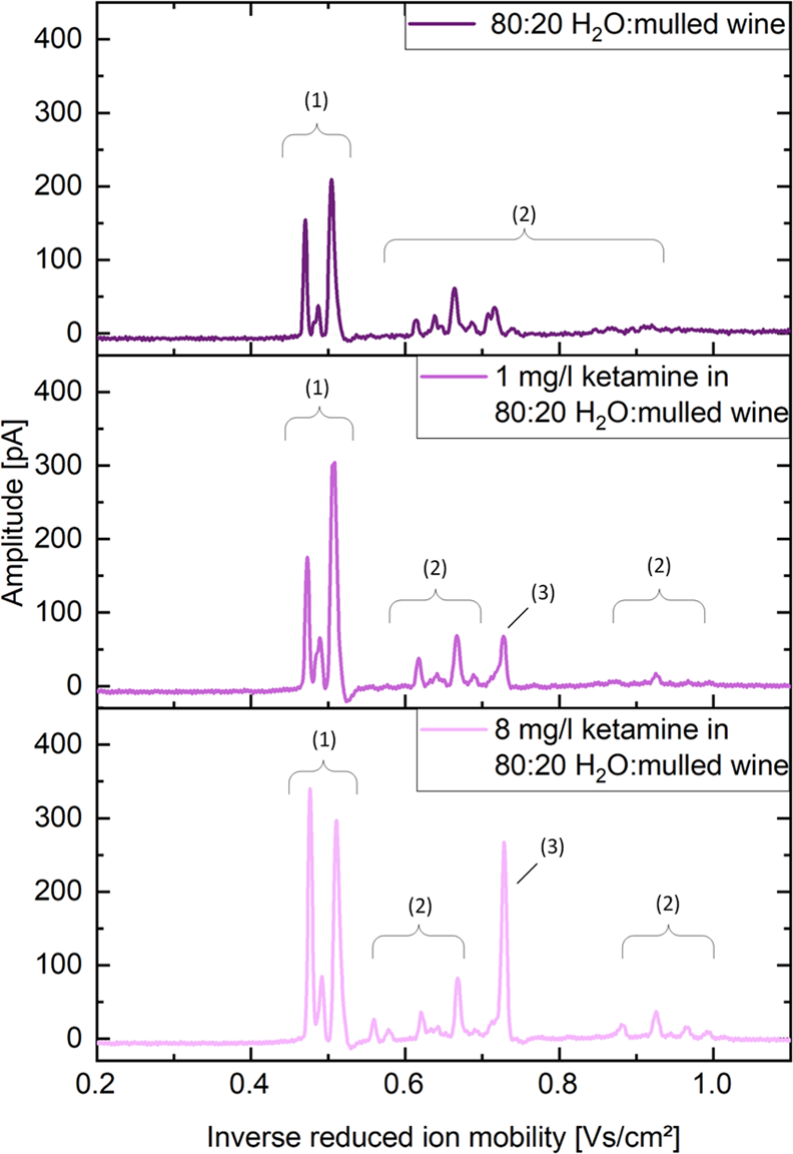
Ion mobility spectra of pure 80:20 water/mulled wine (top) and spiked with 1 mg/L ketamine (middle) and 8 mg/L ketamine (bottom), extracted via coated blades and detected via CBS-IMS. The peaks in the drift time spectrum can be assigned as follows: solvent peaks (1) at *K*_0_ = 2.11, 2.04, and 1.97 cm^2^ V^–1^ s^–1^ and background peaks (2) between *K*_0_ = 1.79 and 1.01 cm^2^ V^–1^ s^–1^ as well as the ketamine peak (3) at *K*_0_ = 1.37 cm^2^ V^–1^ s^–1^.

To investigate the CBS-IMS performance for more complex samples, two exemplary applications have been chosen. First, ketamine was used as an analyte in a water–mulled wine mixture with 0, 1, and 8 mg/L ketamine; see Figure 6. The ketamine peak has a reduced ion mobility of *K*_0_ = 1.37 cm^2^ V^–1^ s^–1^. As shown in Figure 6, besides the solvent peaks at *K*_0_ = 2.11 and 2.04 and *K*_0_ = 1.97 cm^2^ V^–1^ s^–1^, the background of the water–mulled wine mixture also shows smaller peaks between *K*_0_ = 1.79 and 1.01 cm^2^ V^–1^ s^–1^. However, at a concentration of 1 mg/L, a peak associated with ketamine becomes visible at *K*_0_ = 1.37 cm^2^ V^–1^ s^–1^. At a concentration of 8 mg/L, the peak is clearly visible and distinguishable from the background.

For real applications, it is important to know that the detection limit of a substance strongly depends on the matrix. In mulled wine, only concentrations from 1 mg/L can be detected due to the background, while the detection limit in methanol and water is below 1.7 μg/L.

However, the literature indicates that such concentrations of ketamine are not significantly affecting humans compared to knockout drops when taken orally.^38^ In fact, much larger quantities ranging from 100 to 500 mg are typically required for psychoactive effects.^38^ Since CBS-IMS can detect much smaller concentrations, it is well suited for the detection of ketamine in drinks, for example.

**Figure 7:**
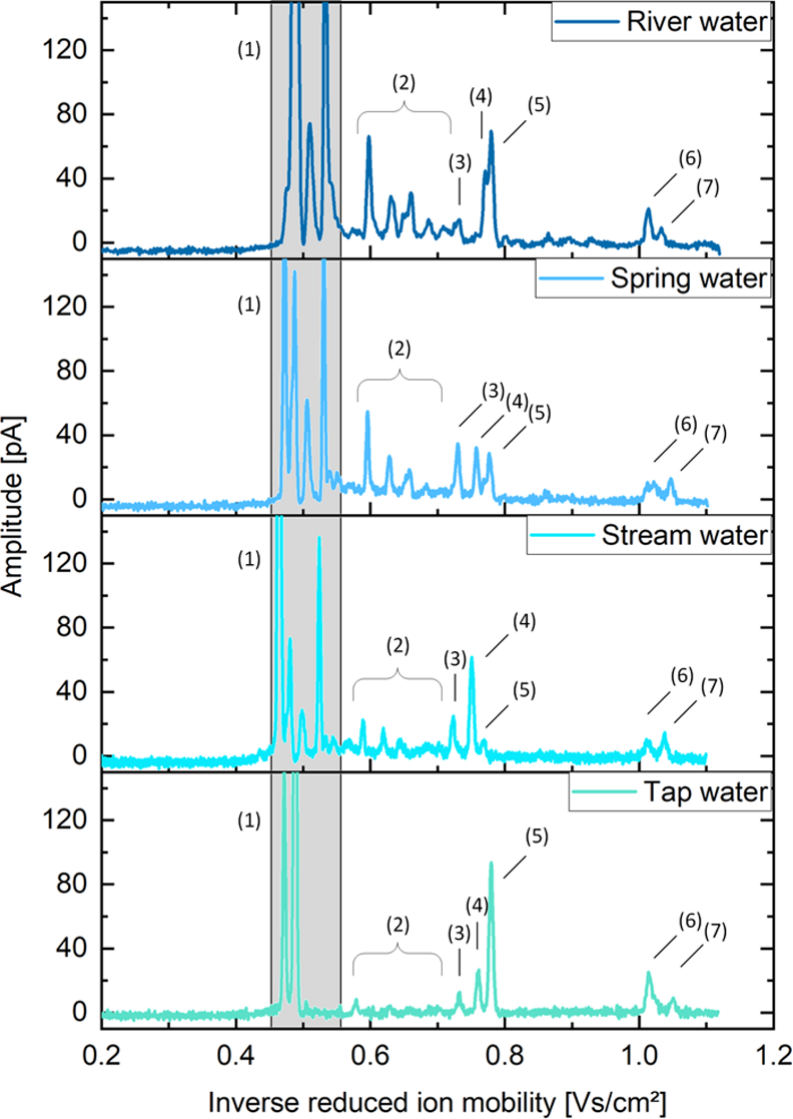
Ion mobility spectra of different types of water spiked with 500 μg/L isoproturon extracted via coated blades and detected via CBS-IMS. The peaks in the ion mobility spectrum can be assigned as follows: solvent peaks (1) between *K*_0_ = 2.12 and 1.82 cm^2^ V^–1^ s^–1^ and background peaks (2) between *K*_0_ = 1.76 and 1.41 cm^2^ V^–1^ s^–1^ as well as the protonated isoproturon monomer peak (3) at *K*_0_ = 1.37 cm^2^ V^–1^ s^–1^, sodium-bound isoproturon monomer (4) at *K*_0_ = 1.32 cm^2^ V^–1^ s^–1^, and an unknown isoproturon monomer adduct (5) at *K*_0_ = 1.29 cm V^–1^ s^–1^ as well as the protonated isoproturon dimer (6) at *K*_0_ = 0.99 cm^2^ V^–1^ s^–1^ and sodium-bound isoproturon dimer (7) at *K*_0_ = 0.96 cm^2^ V^–1^ s^–1^.

Second, we spiked the herbicide isoproturon in various water samples (river water, spring water, stream water, tap water); see Figure 7. Although the water samples have different matrices, resulting in different solvent peaks between *K*_0_ = 2.12 and 1.82 cm^2^ V^–1^ s^–1^ and background peaks between *K*_0_ = 1.76 and 1.41 cm^2^ V^–1^ s^–1^, the peaks of isoproturon can be clearly detected in all samples. In addition to the unknown isoproturon monomer adduct at *K*_0_ = 1.29 cm^2^ V^–1^ s^–1^, the protonated monomer at *K*_0_ = 1.37 cm^2^ V^–1^ s^–1^ and dimer at *K*_0_ = 0.99 cm^2^ V^–1^ s^–1^ as well as the sodium-bound monomer *K*_0_ = 1.32 cm^2^ V^–1^ s^–1^ and dimer at *K*_0_ = 0.96 cm^2^ V^–1^ s^–1^ are also present, as it was in previous ESI-IMS measurements.^37^ As shown in this and other previous publication, an appropriate additive could lead to only one peak per substance.^37,39−41^ Thus, an easier assignment of the analyte peaks in the ion mobility spectrum becomes possible. It proves that especially, the analytes of interest are enriched in the coating of the blades. Larger impurities do not accumulate and are washed away during the rinsing process. This means that CBS provides a useful sample preparation method for IMS. A wide range of practical applications are possible for CBS-IMS, even in complex samples.

## Conclusions

In this paper, the first coupling of coated blade spray and ion mobility spectrometry is presented, and basic feasibility is shown by preliminary investigations. The coated blades allow easy sampling and selective analyte enrichment by microextraction, while the blades also serve as the electrospray ionization emitter. The blades are automatically positioned by an autosampler in front of a compact high-resolution ion mobility spectrometer and wetted with a solvent droplet of 20 μL. This approach exploits the advantages of CBS toward field applications.

It was shown that the individual components can be extracted from a mixture of benzodiazepines and ketamine and that these components can be ionized, separated according to their ion mobility, and detected by IMS. Furthermore, the application in complex matrices such as river water or mulled wine is demonstrated. This shows basic feasibility toward fields of application, for example, in environmental sensing or for point-of-care analysis. To further increase sensitivity, longer extraction times and higher drift voltages can be selected.^28^ Furthermore, the ionization efficiency might be further improved by suitable additives, as has been shown in previous publications on ESI-IMS and ESI-MS.^37,39−41^ Further improving CBS-IMS and investigating additives in the desorption solution for better desorption and ionization efficiency will be carried out in subsequent work.

## References

[ref1] Eiceman G. A.; Karpas Z.; Hill H. H. Ion Mobility Spectrometry, 3rd ed.; CRC Press: Boca Raton, 2013.

[ref2] Chantipmanee N.; Hauser P. C. Development of simple drift tube design for ion mobility spectrometry based on flexible printed circuit board material. Anal. Chim. Acta 2021, 1170, 338626 10.1016/j.aca.2021.338626. 34090588

[ref3] Reinecke T.; Clowers B. H. Implementation of a flexible, open-source platform for ion mobility spectrometry. HardwareX 2018, 4, e00030 10.1016/j.ohx.2018.e00030.

[ref4] Ahrens A.; Möhle J.; Hitzemann M.; Zimmermann S. Novel ion drift tube for high-performance ion mobility spectrometers based on a composite material. Int. J. Ion Mobility Spectrom. 2020, 23, 75–81. 10.1007/s12127-020-00265-0.

[ref5] Drees C.; Höving S.; Vautz W.; Franzke J.; Brandt S. 3D-printing of a complete modular ion mobility spectrometer. Mater. Today 2021, 44, 58–68. 10.1016/j.mattod.2020.10.033.

[ref6] Hauck B. C.; Ruprecht B. R.; Riley P. C. Accurate and on-demand chemical sensors: A print-in-place ion mobility spectrometer. Sens. Actuators, B 2022, 362, 131791 10.1016/j.snb.2022.131791.

[ref7] Thoben C.; Werres T.; Henning I.; Simon P. R.; Zimmermann S.; Schmidt T. C.; Teutenberg T. Towards a miniaturized on-site nano-high performance liquid chromatography electrospray ionization ion mobility spectrometer with online enrichment. Green Anal. Chem. 2022, 1, 100011 10.1016/j.greeac.2022.100011.

[ref8] Hädener M.; Kamrath M. Z.; Weinmann W.; Groessl M. High-Resolution Ion Mobility Spectrometry for Rapid Cannabis Potency Testing. Anal. Chem. 2018, 90, 8764–8768. 10.1021/acs.analchem.8b02180. 29943977

[ref9] Schaefer C.; Lippmann M.; Beukers M.; Beijer N.; van de Kamp B.; Knotter J.; Zimmermann S. Detection of Triacetone Triperoxide by High Kinetic Energy Ion Mobility Spectrometry. Anal. Chem. 2023, 95, 17099–17107. 10.1021/acs.analchem.3c04101. 37946366 PMC10666079

[ref10] Zühlke M.; Riebe D.; Beitz T.; Löhmannsröben H.-G.; Andreotti S.; Reinert K.; Zenichowski K.; Diener M. High-performance liquid chromatography with electrospray ionization ion mobility spectrometry: Characterization, data management, and applications. J. Sep. Sci. 2016, 39, 4756–4764. 10.1002/jssc.201600749. 27805770

[ref11] Kebarle P.; Verkerk U. H. Electrospray: from ions in solution to ions in the gas phase, what we know now. Mass Spectrom. Rev. 2009, 28, 898–917. 10.1002/mas.20247. 19551695

[ref12] Cech N. B.; Enke C. G. Selectivity in Electrospray Ionization Mass Spectrometry. In Electrospray and MALDI Mass Spectrometry, 2nd ed.; Cole R. B., Ed.; John Wiley & Sons, Inc.: Hoboken, NJ, 2010.

[ref13] Zhou S.; Hamburger M. Effects of solvent composition on molecular ion response in electrospray mass spectrometry: Investigation of the ionization processes. Rapid Commun. Mass Spectrom. 1995, 9, 1516–1521. 10.1002/rcm.1290091511.

[ref14] Reinecke T.; Kirk A. T.; Ahrens A.; Raddatz C.-R.; Thoben C.; Zimmermann S. A compact high resolution electrospray ionization ion mobility spectrometer. Talanta 2016, 150, 1–6. 10.1016/j.talanta.2015.12.006. 26838374

[ref15] Piendl S. K.; Raddatz C.-R.; Hartner N. T.; Thoben C.; Warias R.; Zimmermann S.; Belder D. 2D in Seconds: Coupling of Chip-HPLC with Ion Mobility Spectrometry. Anal. Chem. 2019, 91, 7613–7620. 10.1021/acs.analchem.9b00302. 31082255

[ref16] Sukumar H.; Stone J. A.; Nishiyama T.; Yuan C.; Eiceman G. A. Paper spray ionization with ion mobility spectrometry at ambient pressure. Int. J. Ion Mobility. Spectrom. 2011, 14, 51–59. 10.1007/s12127-011-0069-6.

[ref17] Li M.; Zhang J.; Jiang J.; Zhang J.; Gao J.; Qiao X. Rapid, in situ detection of cocaine residues based on paper spray ionization coupled with ion mobility spectrometry. Analyst 2014, 139, 1687–1691. 10.1039/c3an02198j. 24563903

[ref18] Zargar T.; Khayamian T.; Jafari M. T. Immobilized aptamer paper spray ionization source for ion mobility spectrometry. J. Pharm. Biomed. Anal. 2017, 132, 232–237. 10.1016/j.jpba.2016.10.014. 27770685

[ref19] Zargar T.; Khayamian T.; Jafari M. T. Aptamer-modified carbon nanomaterial based sorption coupled to paper spray ion mobility spectrometry for highly sensitive and selective determination of methamphetamine. Microchim. Acta 2018, 185, 103 10.1007/s00604-017-2623-3. 29594391

[ref20] Zarejousheghani M.; Schrader S.; Möder M.; Mayer T.; Borsdorf H. Negative electrospray ionization ion mobility spectrometry combined with paper-based molecular imprinted polymer disks: A novel approach for rapid target screening of trace organic compounds in water samples. Talanta 2018, 190, 47–54. 10.1016/j.talanta.2018.07.076. 30172535

[ref21] Espy R. D.; Muliadi A. R.; Ouyang Z.; Cooks R. G. Spray mechanism in paper spray ionization. Int. J. Mass Spectrom. 2012, 325–327, 167–171. 10.1016/j.ijms.2012.06.017.

[ref22] Gómez-Ríos G. A.; Pawliszyn J. Development of coated blade spray ionization mass spectrometry for the quantitation of target analytes present in complex matrices. Angew. Chem., Int. Ed. 2014, 53, 14503–14507. 10.1002/anie.201407057. 25384839

[ref23] Mirnaghi F. S.; Pawliszyn J. Reusable solid-phase microextraction coating for direct immersion whole-blood analysis and extracted blood spot sampling coupled with liquid chromatography-tandem mass spectrometry and direct analysis in real time-tandem mass spectrometry. Anal. Chem. 2012, 84, 8301–8309. 10.1021/ac3018229. 22928515

[ref24] Poole J. J.; Gómez-Ríos G. A.; Boyaci E.; Reyes-Garcés N.; Pawliszyn J. Rapid and Concomitant Analysis of Pharmaceuticals in Treated Wastewater by Coated Blade Spray Mass Spectrometry. Environ. Sci. Technol. 2017, 51, 12566–12572. 10.1021/acs.est.7b03867. 28990769

[ref25] Tascon M.; Gómez-Ríos G. A.; Reyes-Garcés N.; Poole J.; Boyacı E.; Pawliszyn J. High-Throughput Screening and Quantitation of Target Compounds in Biofluids by Coated Blade Spray-Mass Spectrometry. Anal. Chem. 2017, 89, 8421–8428. 10.1021/acs.analchem.7b01877. 28715206

[ref26] Pawliszyn J. Solid-Phase Microextraction in Perspective. InHandbook of Solid Phase Microextraction; Pawliszyn J., Ed.; Elsevier: Oxford, 2012, Chapter 1.

[ref27] Reyes-Garcés N.; Gionfriddo E.; Gómez-Ríos G. A.; Alam M. N.; Boyacı E.; Bojko B.; Singh V.; Grandy J.; Pawliszyn J. Advances in Solid Phase Microextraction and Perspective on Future Directions. Anal. Chem. 2018, 90, 302–360. 10.1021/acs.analchem.7b04502. 29116756

[ref28] Thoben C.; Raddatz C.-R.; Tataroglu A.; Kobelt T.; Zimmermann S. How to Improve the Resolving Power of Compact Electrospray Ionization Ion Mobility Spectrometers. Anal. Chem. 2023, 95, 8277–8283. 10.1021/acs.analchem.3c00471. 37192335 PMC10233573

[ref29] Thoben C.; Raddatz C.-R.; Lippmann M.; Salehimoghaddam Z.; Zimmermann S. Electrospray ionization ion mobility spectrometer with new tristate ion gating for improved sensitivity for compounds with lower ion mobility. Talanta 2021, 233, 122579 10.1016/j.talanta.2021.122579. 34215071

[ref30] Gabelica V.; Shvartsburg A. A.; Afonso C.; Barran P.; Benesch J. L. P.; Bleiholder C.; Bowers M. T.; Bilbao A.; Bush M. F.; Campbell J. L.; Campuzano I. D. G.; Causon T.; Clowers B. H.; Creaser C. S.; Pauw E. de.; Far J.; Fernandez-Lima F.; Fjeldsted J. C.; Giles K.; Groessl M.; Hogan C. J.; Hann S.; Kim H. I.; Kurulugama R. T.; May J. C.; McLean J. A.; Pagel K.; Richardson K.; Ridgeway M. E.; Rosu F.; Sobott F.; Thalassinos K.; Valentine S. J.; Wyttenbach T. Recommendations for reporting ion mobility Mass Spectrometry measurements. Mass Spectrom. Rev. 2019, 38, 291–320. 10.1002/mas.21585. 30707468 PMC6618043

[ref31] Siems W. F.; Wu C.; Tarver E. E.; Hill H. H. Jr.; Larsen P. R.; McMinn D. G. Measuring the Resolving Power of Ion Mobility Spectrometers. Anal. Chem. 1994, 66, 4195–4201. 10.1021/ac00095a014.

[ref32] Kirk A. T.; Bohnhorst A.; Raddatz C.-R.; Allers M.; Zimmermann S. Ultra-high-resolution ion mobility spectrometry-current instrumentation, limitations, and future developments. Anal. Bioanal. Chem. 2019, 411, 6229–6246. 10.1007/s00216-019-01807-0. 30957205

[ref33] Shvartsburg A. A.; Smith R. D. Ultrahigh-resolution differential ion mobility spectrometry using extended separation times. Anal. Chem. 2011, 83, 23–29. 10.1021/ac102689p. 21117630 PMC3012152

[ref34] Dodds J. N.; May J. C.; McLean J. A. Correlating Resolving Power, Resolution, and Collision Cross Section: Unifying Cross-Platform Assessment of Separation Efficiency in Ion Mobility Spectrometry. Anal. Chem. 2017, 89, 12176–12184. 10.1021/acs.analchem.7b02827. 29039942 PMC5744666

[ref35] Lippmann M.; Kirk A. T.; Hitzemann M.; Zimmermann S. IMS Instrumentation I: Isolated data acquisition for ion mobility spectrometers with grounded ion sources. Int. J. Ion Mobility Spectrom. 2020, 23, 69–74. 10.1007/s12127-020-00260-5.

[ref36] Cochems P.; Kirk A. T.; Zimmermann S. In-circuit-measurement of parasitic elements in high gain high bandwidth low noise transimpedance amplifiers. Rev. Sci. Instrum. 2014, 85, 124703 10.1063/1.4902854. 25554310

[ref37] Thoben C.; Hartner N. T.; Hitzemann M.; Raddatz C.-R.; Eckermann M.; Belder D.; Zimmermann S. Regarding the Influence of Additives and Additional Plasma-Induced Chemical Ionization on Adduct Formation in ESI/IMS/MS. J. Am. Soc. Mass Spectrom. 2023, 34, 857–868. 10.1021/jasms.2c00348. 37052511 PMC10161231

[ref38] Zanos P.; Moaddel R.; Morris P. J.; Riggs L. M.; Highland J. N.; Georgiou P.; Pereira E. F. R.; Albuquerque E. X.; Thomas C. J.; Zarate C. A.; Gould T. D. Ketamine and Ketamine Metabolite Pharmacology: Insights into Therapeutic Mechanisms. Pharmacol. Rev. 2018, 70, 621–660. 10.1124/pr.117.015198. 29945898 PMC6020109

[ref39] Yang X. J.; Qu Y.; Yuan Q.; Wan P.; Du Z.; Chen D.; Wong C. Effect of ammonium on liquid- and gas-phase protonation and deprotonation in electrospray ionization mass spectrometry. Analyst 2013, 138, 659–665. 10.1039/C2AN36022E. 23181258

[ref40] Mortier K. A.; Zhang G.-F.; van Peteghem C. H.; Lambert W. E. Adduct formation in quantitative bioanalysis: effect of ionization conditions on paclitaxel. J. Am. Soc. Mass Spectrom. 2004, 15, 585–592. 10.1016/j.jasms.2003.12.013. 15047063

[ref41] Kruve A.; Kaupmees K. Adduct Formation in ESI/MS by Mobile Phase Additives. J. Am. Soc. Mass Spectrom. 2017, 28, 887–894. 10.1007/s13361-017-1626-y. 28299714

